# Family Planning Supply Environment in Kinshasa, DRC: Survey Findings and Their Value in Advancing Family Planning Programming

**DOI:** 10.9745/GHSP-D-15-00298

**Published:** 2015-12-15

**Authors:** Patrick Kayembe, Saleh Babazadeh, Nelly Dikamba, Pierre Akilimali, Julie Hernandez, Arsene Binanga, Jane T Bertrand

**Affiliations:** ^a^​ University of Kinshasa, School of Public Health, Kinshasa, Democratic Republic of Congo; ^b^​ Tulane School of Public Health and Tropical Medicine, Department of Global Health Management and Policy, New Orleans, LA, USA; ^c^​ Tulane International LLC, Kinshasa, Democratic Republic of Congo

## Abstract

**Background:**

Modern contraceptive prevalence was 14.1% in 2007 in Kinshasa, the capital city of the Democratic Republic of the Congo (DRC). Yet virtually nothing was known about the family planning supply environment.

**Methods:**

Three surveys of health facilities were conducted in 2012, 2013, and 2014 to determine the number, spatial distribution, and attributes of sites providing family planning services. The 2012 and 2013 surveys aimed to identify the universe of family planning facilities while obtaining a limited set of data on “readiness” to provide family planning services (defined as having at least 3 modern methods, at least 1 person training in family planning in the last 3 years, and an information system to track distribution of products to clients) and output (measured by couple-years of protection, or CYP). In contrast, the 2014 survey, conducted under the umbrella of the Performance Monitoring and Accountability 2020 (PMA2020) project, was based on 2-stage cluster sampling. This article provides detailed analysis of the 2012 and 2013 surveys, including bivariate and multivariate analysis of correlates of readiness to provide services and of output.

**Results:**

We identified 184 health facilities that reported providing at least 1 contraceptive method in 2012 and 395 facilities in 2013. The percentage of sites defined as “ready” to provide services increased from 44.1% in 2012 to 63.3% in 2013. For the 3-month period between January and March 2013, facilities distributed between 0 and 879.2 CYP (mean, 39.7). Nearly half (49%) of the CYP was attributable to implants, followed by IUDs (24%), CycleBeads (11%), and injectables (8%). In 2013, facilities supported by PEPFAR (n = 121) were more likely than other facilities to be rated as ready to provide services (*P*<.0001); however, PEPFAR-supported sites generated less CYP on average than sites supported by family planning implementing agencies (*P*<.0001). Multivariate analysis showed 3 variables were associated with CYP: type of health facility, length of time in operation, and number of contraceptive methods available. Clinics generated higher (3-month) CYP than hospitals and health centers by 65.3 and 61.5 units, respectively (*P*<.01). The mean CYP for facilities in operation for 4–6 years was 26.9 units higher (*P*<.05), and 50.2 units higher for those operating 7+ years (*P*<.01), than the reference group of facilities in operation for 1 year or less. For each additional method available at a facility, CYP increased by almost 8 units (*P*<.01).

**Conclusions:**

Findings from these surveys suggest that lack of physical access is not the defining reason for low contraceptive use in Kinshasa, although it is highly likely that other service-related factors contribute to low service utilization. The results contributed to increasing the momentum for family planning in the DRC in many ways, including mobilizing partners to increase contraceptive access and increasing donor investment in family planning in the DRC.

## Abstract (precis)

A series of facility-based surveys that mapped all sites providing family planning services and that assessed readiness to provide services, using mobile phones, was feasible in a low-resource setting, contributing to mobilization of partners and increased donor support. Between 2012 and 2013, readiness to provide services increased from 44% of sites to 63%. Three factors most associated with productivity: type of facility (clinics more than hospitals or health centers), more years in operation, and number of methods available.

## INTRODUCTION

Kinshasa, the capital city of the Democratic Republic of the Congo (DRC), has a population of approximately 10 million people. The DRC is typical of many sub-Saharan African countries with a high total fertility rate (6.6), although it is 4.2 in Kinshasa.^1^ Family planning efforts commenced in the country in the 1980s, but the DRC government did not begin to publicly demonstrate commitment to family planning until 2012.^2^ At that time, a number of government agencies and nongovernmental organizations (NGOs) were working in isolation to provide family planning services in different locations throughout the sprawling city of Kinshasa, but there was no master plan or even centralized listing of service delivery points (SDPs). In short, Kinshasa was a virtual black box in terms of the family planning service delivery environment.^3^

Beginning in 2012, the Kinshasa School of Public Health in collaboration with the Tulane School of Public Health and Tropical Medicine conducted research to assess the availability of contraceptives in health facilities and other aspects of the family planning supply environment throughout Kinshasa. The latest Demographic and Health Survey (DHS) at that time (from 2007) showed the modern contraceptive prevalence rate (mCPR) in Kinshasa to be only 14.1%, a percentage similar to other capital cities in francophone Africa.^4^ (Surveys since then have shown an increase in mCPR in Kinshasa—to 20.4% in the Performance Monitoring and Accountability 2020 [PMA2020] survey of 2014.^5^) This finding begged the question: was low mCPR an issue of supply (i.e., limited availability of contraceptive methods) or demand (lack of interest in using contraception)? The 2007 DHS indicated unmet need for family planning among married women in Kinshasa was 26.9% (19.9% unmet need for spacing births and 7.0% for limiting births). No comparable data existed on contraceptive availability.

The organization of family planning services in Kinshasa contrasts markedly to countries in which the government manages family planning services through a network of public sector health facilities. In the DRC, the central government supplies only 15% of the national health budget; donors contribute 23%, international NGOs 11%, corporations 8%, and the remaining 43% constitutes out-of-pocket payments, although even then the actual disbursement of funds is lower than the amount budgeted.^6^ In family planning as in other health sectors, the government pays for personnel and infrastructure while donor funding covers the vast majority of programmatic expenses, including training, commodities, behavior change communication, and related activities. Data from the National Health Account indicate that for reproductive health, the government covers less than 1% of the costs, donors 31%, international NGOs 1%, and households the remaining 68%.^6^ Donor funding in the DRC is channeled through international and local NGOs that provide family planning services through their own health facilities or that support family planning and reproductive health services in government facilities. As a result, the distinction between public and private sector facilities is often blurred.

The National Reproductive Health Program (Programme National de Reproduction de la Santé, PNSR) is mandated to establish national service delivery norms and oversee family planning activity throughout the country, yet for many years it lacked the financial, technical, and human resources to effectively advance family planning programming. Prior to 2012, it worked in loose collaboration with different implementing partner organizations (NGOs receiving donor funding to provide family planning services in Kinshasa and/or elsewhere in the country). As of 2012, 9 partner organizations supported family planning services in one or more of the 35 health zones of Kinshasa (Box). These NGOs generally coordinated with the Médecin Chef de Zone (chief medical officer of the zone), but no centralized database existed with the number or location of family planning SDPs in Kinshasa.

In June 2012, another source of support became available for family planning in Kinshasa: 4 projects supported by the US President's Emergency Plan for AIDS Relief (PEPFAR) began to introduce contraception (counseling and methods) into their PMTCT (prevention of mother-to-child transmission) services. PEPFAR supported the training, and the US Agency for International Development (USAID), as an implementing arm of PEPFAR, supplied the full range of contraceptives. In 2013, PEPFAR declared family planning to be the second of four pillars for PMTCT,^7^ and by mid-2013, PEPFAR had integrated family planning into a second wave of facilities. Although the DRC has relatively low HIV prevalence in comparison with East and Southern African countries, it receives PEPFAR funding for HIV prevention and treatment. In mid-2012, 4 US government-funded projects (Box) expanded their PMTCT work to include family planning.^8^ Specifically, the projects identified health facilities where they could train personnel in contraceptive service delivery and supply them with commodities.

An essential first step to understanding contraceptive availability and eventually strengthening family planning programming was to determine the number and location of family planning SDPs in Kinshasa, a challenging task in a city of 10 million people that covers a landmass the size of the country of Lebanon. This initiative evolved into a series of 3 family planning facility-based surveys (in 2012, 2013, and 2014). Although the surveys differed in content, data collection mechanism, and sampling techniques, they have produced a wealth of data to inform programmatic decision making and to assess progress in family planning service delivery. In this paper, we describe results of the surveys, focusing on the 2012 and 2013 surveys in particular (as their methodology was more comparable than that of the 2014 survey), to demonstrate contraceptive availability, readiness to deliver family planning services, and performance of the facilities (level of output), as well as correlates of readiness to provide services and of output. In addition, the survey results provide information on the contribution of PEPFAR-supported sites to the family planning service delivery environment.

A series of facility-based surveys was conducted in Kinshasa, DRC, to explore the family planning supply environment.

**BOX. Organizations Providing Family Planning Services in Kinshasa**

**As of 2012:**

1. Association Bien-être de la Famille (ABEF), member association of the International Planned Parenthood Federation (IPPF)
2. Association de Santé Familiale (the local affiliate of Population Services International, PSI)
3. Conduite de la Fécondité (Catholic organization promoting natural methods)
4. DKT International
5. Handicap International (a UK-based NGO)
6. Maman An’Sar (a Muslim organization that promotes family planning)
7. PARSS, World Bank project for integrated health services delivery, which closed in 2014
8. PASSKIN, initiative of Canadian Cooperation implemented by the Centre de Coopération Internationale en Santé et Développement (CCISD), which ended in 2014
9. Programme National de la Santé de la Reproduction (PNSR), Ministry of Health

**Added in mid-2012 (with PEPFAR funding in the DRC):**

1. Elizabeth Glaser Pediatric AIDS Foundation (EGPAF)
2. ICAP (a Columbia University initiative)
3. ProVIC (DRC Integrated HIV/AIDS Project)
4. University of North Carolina (UNC)

## METHODOLOGY

### Study Design and Sampling

We conducted facility-based surveys in Kinshasa in 2012, 2013, and 2014. (Data were also collected in 2015 but had not been officially released at the time of writing of this article.) See Table 1 for a summary of the methodological approach to each survey.

**TABLE 1:** Methodological Approaches to the 3 Facility-Based Surveys in Kinshasa, DRC

	2012	2013	2014
Dates of data collection	Jan–Mar 2012	Oct 2013–Jan 2014	Aug–Sep 2014
Approach to sampling of sites	Attempted to identify universe of family planning sites	Attempted to identify universe of family planning sites	Random sample of 58 enumeration areas; up to 6 SDPs per enumeration area
Type of facilities included	Health facilities (hospital, health center, health post, clinic) and commercial pharmacies	Health facilities (hospital, health center, health post, clinic); no pharmacies	Up to one each per enumeration area:• hospital• health center• health post• clinic• pharmacy• kiosk
Mechanism for data collection	Pencil and paper	Smartphone	Smartphone
Length of questionnaire	Short	Short	Detailed
Used 3-star “readiness” rating	Yes	Yes	No
Geocoding of SDPs	Yes	Yes	Yes

Abbreviations: DRC, Democratic Republic of the Congo; SDP, service delivery point.

In 2012, we conducted a facility-based survey to identify, survey, and geocode the universe of health facilities and pharmacies that sold or distributed contraception free of charge, as a first step in defining the family planning supply environment in Kinshasa. Although the primary focus was on health facilities, we added pharmacies because they represented the source of contraception for almost half of modern contraceptive users in the 2007 DHS.^4^ To identify all possible health facilities, we drew up lists for each health zone, based on information obtained from the PNSR and implementing partners. Once at the health zone, we completed our listings with information provided by the health zone authorities. Data were collected using the conventional paper and pencil method; geographical coordinates were taken with an eTrex global positioning system (GPS) device using the WGS84 reference coordinate system.

In 2013, we conducted a follow-up survey using the same methodological approach as in 2012. The 2013 survey had 2 key objectives: (1) to update information on the universe of family planning sites, and (2) to evaluate the change in the percentage of 3-star sites (explained below). This survey did not include pharmacies because we discovered that the vast majority of pharmacies are in the private sector operating independently of the PNSR or NGOs working in family planning, and thus they were unlikely to be targets of family planning interventions. Also, the high use of pharmacies identified in the 2007 DHS was linked to the widespread use of condoms, but use of condoms for family planning had decreased from 62% of modern method use in 2007 to 30%–42% between 2013 and 2015, as other methods became available.^1^^,^^5^ We started with the listing of sites from the 2012 survey and added new sites that partner organizations were now supporting (including the PEPFAR-supported projects); we also conferred with health zone authorities. The 2013 survey used Android smartphones and the OpenDataKit application for both data collection and geocoding of sites. This open-source tool was programmed using XML forms tailored to collect family planning service indicators (such as number of trained staff or contraceptive methods available on that day) for each facility. In addition, the internal GPS for the smartphone automatically collected latitude and longitude data, thus recording the exact location of the facility. This innovative technology yielded rapid results (within a month of the completion of data collection), which brought further attention to the survey.

The 2014 survey differed markedly from the previous 2 surveys. Kinshasa was selected as a site for the PMA2020 survey project that uses a mobile-assisted data collection system to monitor family planning programs.^5^ Because the PMA2020 survey would be repeated at least annually in Kinshasa for multiple years, there was great interest in adopting this methodology. Key differences were the sampling approach and content of the questionnaire. Instead of capturing the universe of family planning SDPs, the PMA2020 survey was based on 2-stage cluster sampling, and the data were weighted accordingly. In the first stage, 58 enumeration areas were randomly selected (from the total of 335 in Kinshasa). During the second stage, one each of the following types of facilities was randomly selected per enumeration area: hospital, health center, health post, clinic, pharmacy, and kiosk. The difference between a health center and clinic is based on the level of care the facility offers and on the personnel operating it. A health center offers a minimum package of services, including promotional and curative. A clinic offers more than the minimum package, including specialized consultations, interventions, and hospitalizations. Because not all enumeration areas had all 6 types of facilities, the actual number obtained per enumeration area ranged from 3 to 6. Regarding the content of the questionnaire, the PMA2020 survey included many more variables than the 2012 and 2013 surveys. Smartphones and the ODK system were used for both data collection and geocoding of sites.

### Data Collection

Although the 2012 and 2013 questionnaires were purposely kept very short, they included 3 variables that were used to construct a very simple index of “readiness” to provide family planning services as a rough means of assessing differential levels of capacity among facilities. The 3 variables were:

1. Availability of at least 3 modern contraceptive methods
2. Availability of at least 1 person trained in family planning in the past 3 years
3. Availability of an information system that tracked distribution of products to clients

3 variables were used to construct a simple index of readiness to provide family planning services.

Facilities having all 3 items were labeled “3-star.” In a previous publication by the authors,^2^ we introduced this index as a 3-star rating of quality. However, given that service quality is more complex, we have renamed it “readiness” in this article. Although a crude indicator, it served a useful programmatic purpose of giving implementing partners 3 specific areas for improving service delivery at the sites they supported.

The 2013 survey also collected data on the number of commodities distributed during a 3-month period (January–March 2013) at each site, which were then converted to couple-years of protection (CYP) using established conversion factors.^9^ (CYP data were also collected in the 2012 survey, but the data were not of sufficient quality to include in this analysis.) CYP is a widely used measure of output in international family planning programs. Although it does not track individual users, it reflects the volume of activity at a given site and is useful for comparison purposes.^10^^,^^11^ To obtain a general sense of the distribution of methods provided in 2013, we calculated the percentage of method mix attributable to each method from the CYP data. We recognize the bias of calculating method mix based on CYP, since a method is given full credit for the protection it confers in the year it is delivered. For example, the conversion factor for 1 Jadelle implant is 3.8 years, the average duration of actual use based on research studies. However, the program gets all 3.8 years of credit in the year it is inserted. By contrast, methods whose protection lasts less than 1 year, such as the Depo-Provera injectable, never benefit from this bias. On the other hand, such methods provide a far lower duration of protection than longer-acting methods.

### Data Analysis

Statistical analysis using Stata 13.0 package software consisted of bivariate and multivariate regression of data from the 2013 survey to test for factors as possible correlates of readiness (3-star rating) and of output (CYP). The 4 independent variables tested in the bivariate regression were: type of managing authority (private, faith-based, government, NGO), type of facility (health center, clinic, hospital), number of years in operation, and number of days per week in operation. Multivariate analysis was applied to test the relationship of 8 independent variables to the 3-month CYP. In addition to 4 aforementioned independent variables (type of facility, managing authority, number of years in operation, and number of days per week in operation), we also included type of support for each facility and the 3 variables that make up the readiness rating (number of methods, number of trained staff, and having an information system) in the model. We opted to test the 3 component parts of the readiness index as separate variables in the multivariate analysis to identify which was most strongly related to CYP. We also analyzed the contribution of external support (by a family planning implementing organization or by PEPFAR) to the family planning service delivery environment. Chi-square and *t* test and analysis of variance (ANOVA) were used to test the significance of associations between variables. A *P* value≤.05 was considered as evidence of association between 2 variables.

### Ethics

The 2012 and 2013 facility-based surveys were approved by the Tulane Institutional Review Board (#238734 and #493349, respectively), as well as by the Ethics Committee of the Kinshasa SPH (ESP/CE/043/11 and ESP/CE/072/13).

## RESULTS

### Facilities Offering Contraceptive Methods

In 2012, we identified 184 health facilities (including hospitals, clinics, health centers, and health posts), as well as 1,345 pharmacies, that provided at least 1 contraceptive method. In 2013, we found more than twice the number of health facilities (395) (Table 2). This doubling of sites is due to 3 main factors: addition of new sites by existing implementing partners; addition of sites by new PEPFAR-supported partners; and more intensive efforts by the research team to identify family planning sites within each health zone.

**TABLE 2:** Number and Types of SDPs That Reported Offering Contraception in Kinshasa, DRC, 2012, 2013, and 2014 Facility-Based Surveys^a^

	2012			2013		2014		
SDP Type	No.	% of all health facilities^b^	% of all SDPs^b^	No.	% of all health facilities^b^	No.	% of all health facilities^b^	% of all SDPs^b^
Health facilities (excluding pharmacies)								
Hospital	23	12.5	1.5	72	18.2	17	21.5	10.6
Clinic	8	4.3	0.5	20	5.1	6	7.5	3.8
Health center	151	82.1	9.8	302	76.5	55	68.8	34.4
Heath post	2	1.1	0.1	1	0.3	1	1.3	0.6
**Subtotal**	**184**	**100.0**	**12.0**	**395**	**100.0**	**80**	**100.0**	**50.0**
Pharmacies	1345	–	87.6	–	–	80	–	50.0
**Total SDPs**	**1535**	**–**	**100.0**	**–**	**–**	**160**	**–**	**100.0**

Abbreviations: DRC, Democratic Republic of the Congo; SDP, service delivery point.

a The 2014 survey took a sample of SDPs, whereas the 2012 and 2013 surveys attempted to identify the universe of health facilities providing family planning. The 2013 survey did not include pharmacies.

b That reported offering contraception.

Health centers were the predominant type of health facility that offered family planning services in both 2012 (82.0%) and 2013 (76.5%). The 2014 data—representing the sample rather than the universe of facilities—nonetheless yielded a similar distribution by type of health facility to the previous years. (Data on pharmacies—available in the 2012 and 2014 surveys—are displayed in Table 1 but excluded from the narrative presentation of results, given the focus of this article on the other types of health facilities.)

**FIGURE 1.**
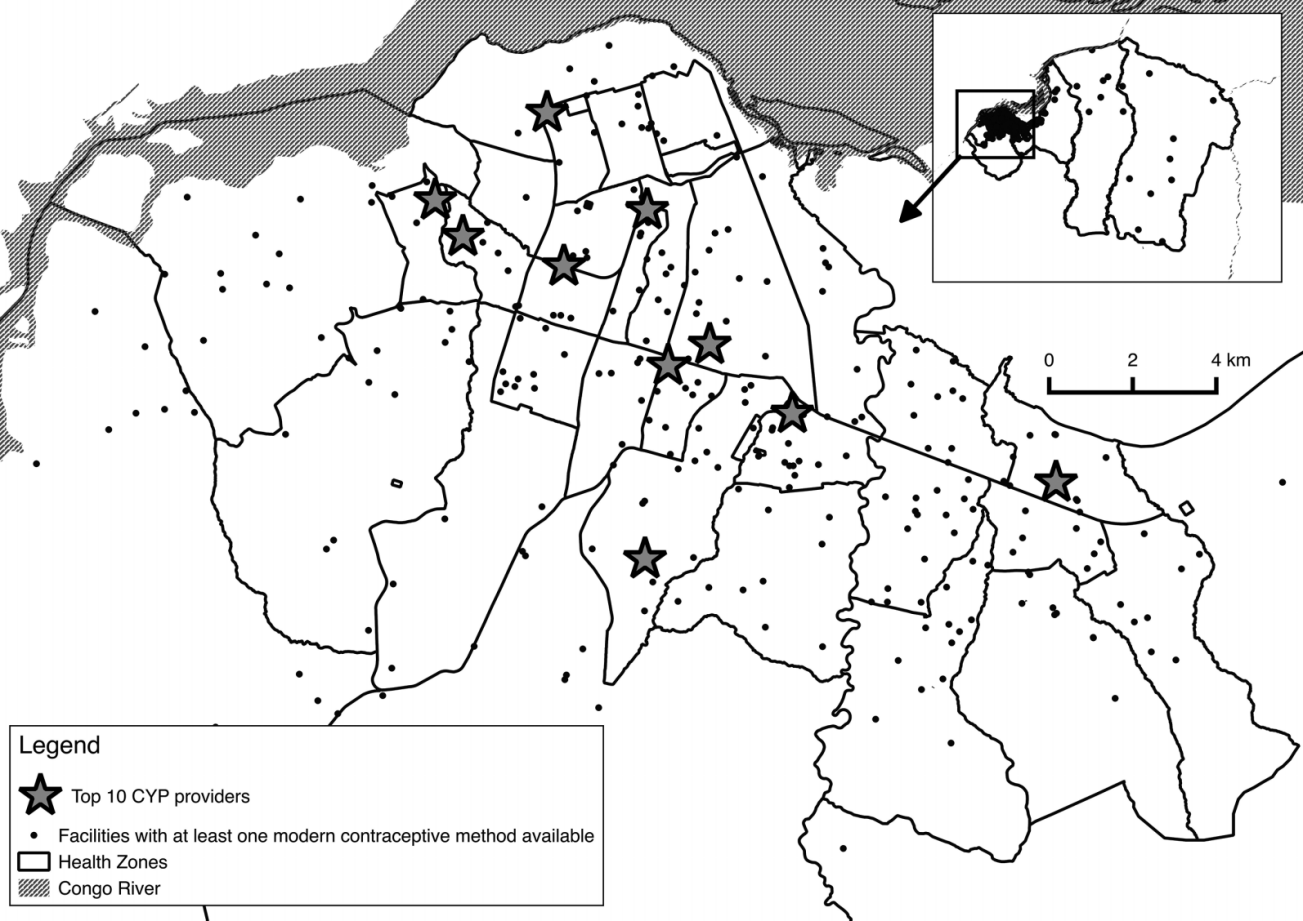
Spatial Distribution of Health Facilities Providing Contraception, Kinshasa, Democratic Republic of the Congo, 2013 Abbreviation: CYP, couple-years of protection.

**FIGURE 2.**
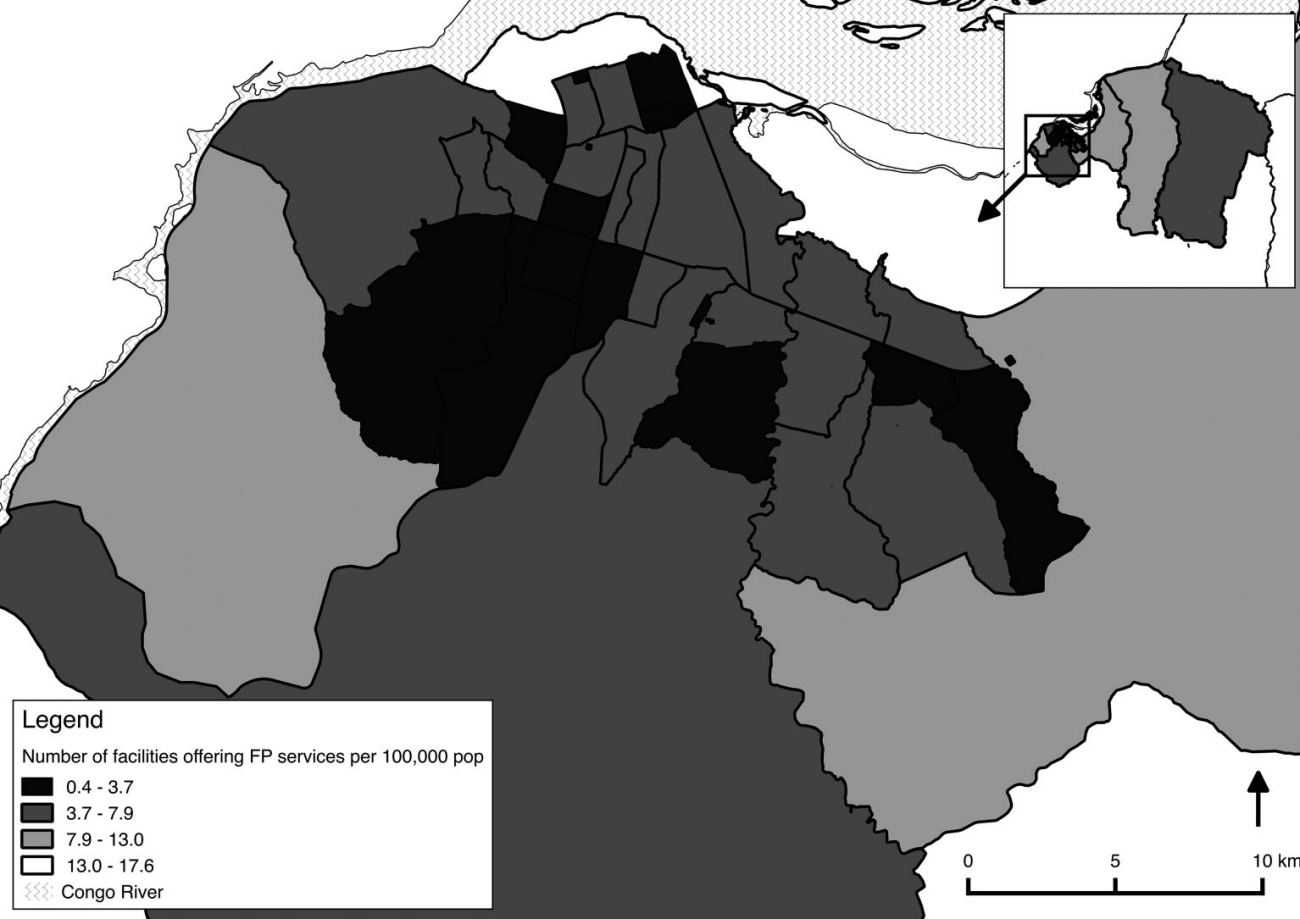
Number of Facilities per Health Zone Offering at Least One Method of Contraception per 100,000 Population, Kinshasa, Democratic Republic of the Congo, 2013

Geocoding of the facilities in all 3 surveys allowed for spatial analysis of the distribution of family planning sites. Figure 1 shows the distribution of health facilities (excluding pharmacies) that were offering at least 1 contraceptive method in 2013. The concentration of facilities is far greater toward the center of Kinshasa than in the peripheral health zones. The number of health facilities that offered at least 1 method of contraception per health zone varied from 4 to 22. Taking into consideration population density, the number of health facilities offering at least 1 method of contraception per 100,000 population varied from 0.38 (Kokolo) to 17.57 (Gombé) per health zone. The map in Figure 2 shows these ratios at the health zone level for the entire city of Kinshasa. In downtown Gombé (the only health zone appearing in white), the high ratio of facilities per 100,000 results in large part from the low population density living in this largely administrative area of the city. In the surrounding health zones, the high population concentration results in a greatly reduced number of facilities offering family planning per 100,000 population. By contrast, in the very large semi-rural health zone located on the eastern periphery, one must take into consideration the distance factor. Although the number of facilities per 100,000 population is higher than in the center of Kinshasa, the vast landmass of these 2 eastern-most health zones means that the distances to the nearest health facility offering contraception can be as far as 25 kilometers for some people.

Facilities were concentrated in the center of Kinshasa than in peripheral health zones.

### Types of Available Contraceptive Methods

All the facility-based surveys yielded information on the number and type of contraceptives available. In the 2012, 2013, and 2014 surveys, at least half the sites had at least 3 modern contraceptive methods available on the day of the visit. Although the rank ordering differed slightly, these methods comprised condoms, injectables, and pills.

At least half the sites had at least 3 modern methods available on the day of the survey.

**FIGURE 3.**
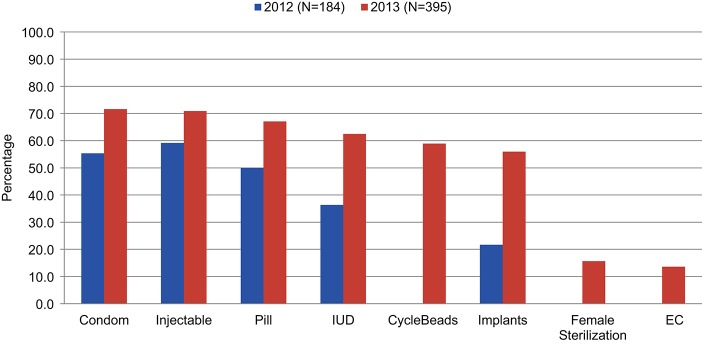
Availability of Specific Contraceptive Methods at Surveyed Facilities, Kinshasa, Democratic Republic of the Congo, 2012 and 2013 Abbreviations: EC, emergency contraception; IUD, intrauterine device. CycleBeads, female sterilization, and EC were not included in the 2012 survey.

Figure 3 presents the availability of specific methods in these health facilities in both 2012 and 2013 (with a different denominator to reflect the larger number of sites identified and surveyed in 2013). For every method measured in both surveys, availability increased between 2012 and 2013. Condoms and injectables were the most frequently available in both years, followed by pills and intrauterine devices (IUDs). By 2013, CycleBeads and implants were also offered in over half the facilities surveyed. Far less available were female sterilization and emergency contraception (16% and 14% of surveyed facilities, respectively, in 2013).

Condoms and injectables were the most frequently available methods in the facilities.

### Readiness to Provide Family Planning Services

As reported elsewhere,^3^ the percentage of 3-star sites (those that had at least 3 modern contraceptive methods available, at least 1 person trained in family planning, and a functioning information system) increased from 44.1% in 2012 to 63.3% in 2013, reflecting measurable progress in readiness to provide contraception, especially considering the doubling in the number of facilities providing family planning between the 2 surveys.

The percentage of sites defined as ready to provide family planning services increased from 44% to 63% between 2012 and 2013.

### Couple-Years of Protection Delivered (Output)

In 2013, the number of CYP distributed per facility for the 3-month period between January and March 2013 ranged from 0 to 879.2. Extrapolating the number of CYP for the 3-month period for the top-performing site to a full 12 months results in 3,517 CYP (879.2 CYP for 3 months = 293.1 CYP per month, multiplied by 12 months = 3,516.8 CYP per year). Some of the CYP was generated by long-acting methods, meaning that the protection from the method (e.g., an implant) would last beyond a single calendar year. Yet this number (3,517) quantifies the protection provided by this 1 facility. In stark contrast, the mean CYP for the 3-month period across the 395 sites was 39.7, equivalent to roughly 158 couples protected in a 1-year period (39.7 CYP for 3 months = 13.2 per month, multiplied by 12 months = 158.4 CYP per year).

Facilities delivered, on average, 39.7 CYP over a 3-month period in 2013

The top 10 facilities in terms of CYP performance represented less than 3% of the total number of sites, but they generated 31% of the total CYP for that period (data not shown in tables). On average, the top 10 facilities had more methods available (5.3) than did other facilities (3.6). The spatial distribution of these top 10 facilities is shown on the map in Figure 1, indicating a spread across the more concentrated population areas in the city (although distant to the outlying health zones).

Table 3 shows the percentage of method mix attributable to each method in 2013, calculated from CYP. Half the CYP corresponded to implants, followed by IUDs (24%), the Standard Days Method/Cyclebeads (11%), and 3-month Depo-Provera injectables (8%). Although the 2015 PMA2020 survey in Kinshasa showed male condoms to be the most frequently used modern method (by 35% of modern method users),^12^ condoms are often purchased in pharmacies or retail outlets, which were not visited in the 2013 facility-based survey.

49% of the CYP delivered in 2013 corresponded to implants, and 24% to IUDs.

**TABLE 3:** Three-Month (January–March 2013) CYP by Method, Kinshasa, Democratic Republic of the Congo

Method	Total CYP	% of Total CYP
Jadelle	6346	48.8
IUD	3100	23.8
CycleBeads	1397	10.7
3-month injectable	1011	7.8
Female sterilization	650	5.0
Implanon	158	1.2
Male condom	152	1.2
Male sterilization	90	0.7
Pill	64	0.5
1-month injectable	21	0.2
Female condom	17	0.1
Emergency contraception	2	0.0
Diaphragm	0	0.0
**Total**	**13,008**	**100.0**

Abbreviations: CYP, couple-years of protection; IUD, intrauterine device.

Of note, one-third (33.4%) of the sites surveyed in 2013 reported *zero* CYP for the period January to March 2013 (that is, no reported distribution of contraceptive methods to clients). Facilities with no information system (78.6%) were more likely to have zero CYP than those with an information system (23.7%). Given the doubling in number of sites between 2012 and 2013, some sites surveyed at the end of 2013 may not have been operational in the beginning of that year during the period when this measure of CYP was taken. However, among 295 health facilities that had been offering family planning for at least 1 year, at least one-quarter (26.1%) reported zero CYP (data not shown).

### Correlates of Readiness to Provide Family Planning Services and of CYP

As shown in Table 4, 2 factors emerged as correlates of readiness in the bivariate analysis: type of facility and hours of operation. Managing authority and number of years in operation were not associated with readiness. Also shown in Table 4, 3 of the 4 tested factors emerged as correlates of performance (CYP output): type of facility, number of days per week in operation, and number of years in operation. It increased monotonically with the number of years the facility had been in operation. And it was higher among facilities open 4 to 6 days a week than among other facilities.

**TABLE 4:** Correlates of Readiness (Based on the 3-Star Rating) and of Output (Based on CYP) in the 2013 Survey, Kinshasa, DRC: Results of Bivariate Regression Analysis

		Readiness: Percentage Rated 3-Star		Output: Mean CYP (3-Month Period)	
	No.	%	*P* Value^a^	Mean	*P* Value^b^
Total	395	63.3		39.7	
Managing authority			.37		.34
Private	120	61.7		26.4	
Faith-based	100	68.0		48.5	
Government	97	57.7		38.7	
NGO	75	68.0		51.3	
Other	3	33.3		22.6	
Type of health facility			.03		.02
Health center	303	59.7		35.5	
Clinic	20	85.0		97.3	
Hospital	72	72.2		41.5	
No. of years in operation			.25		.001
0–1	100	56.0		7.3	
2–3	121	68.9		34.9	
4–6	83	61.9		47.8	
7 or more	89	65.2		62.8	
No. of days/week in operation			.05		.03
0–3	121	54.5		24.6	
4–6	205	66.8		51.5	
7	69	68.1		31.3	

Abbreviations: CYP, couple-years of protection; DRC, Democratic Republic of the Congo.

a *P* value calculated using chi-square test.

b *P* value calculated using ANOVA test.

### The Entry of PEFPAR into Family Planning Service Delivery

The 2013 survey—conducted after PEPFAR had scaled-up its integration of family planning into PMTCT services—identified 121 health facilities supported by PEPFAR that reported providing family planning services. In comparison, 187 sites identified in the 2013 survey were supported by a family planning implementing organization (such as those listed in the Box) and 87 had no external support (Table 5).

**TABLE 5:** Attributes and Performance of Facilities Providing Family Planning Services by Source of External Support, Kinshasa, Democratic Republic of the Congo, 2013

		Source of External Support			
	Total (N = 395)	FP Implementing Organization (n = 187)	PEPFAR (n = 121)	None (n = 87)	*P* Value
No. of years in operation, mean	4.7	5.6	3.0	4.9	.05
No. of days per week of FP service delivery, mean	4.8	4.9	4.7	4.7	.15
Type of facilities, %					
Hospital	72	31	20	21	.05
Health center	302	148	90	65	
Clinic	20	8	11	1	
Achievement of elements in 3-star readiness index, %					
3+ methods	72.9	78.6	92.6	33.3	<.001
Staff trained in FP	88.9	93.6	94.2	71.3	<.001
Basic information system	82.3	90.4	87.6	57.5	<.001
All 3 elements	63.3	71.7	77.7	25.3	<.001
CYP, Jan–Mar 2013, mean	39.7	61.3	23.7	15.8	<.001
Zero CYP (no methods distributed), %	33.4	20.9	43.0	47.1	<.001

Abbreviations: CYP, couple-years of protection; FP, family planning; PEPFAR, US President’s Emergency Plan for AIDS Relief.

**FIGURE 4.**
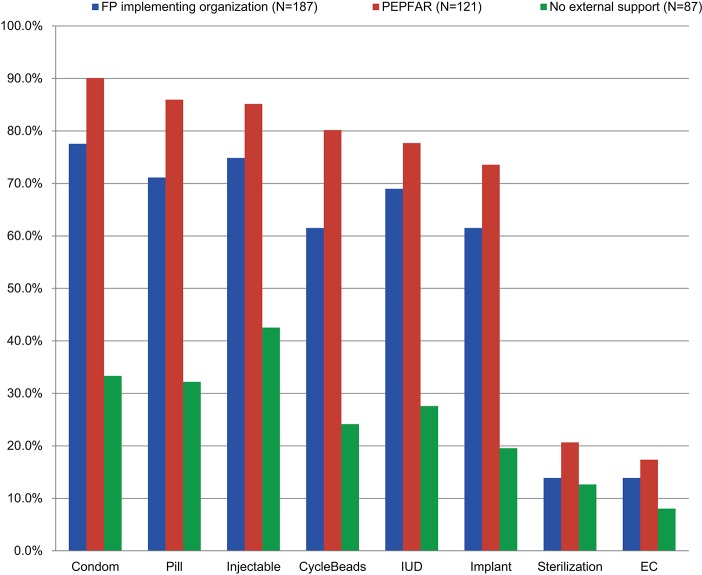
Availability of Specific Contraceptive Methods by Source of External Support, Kinshasa, Democratic Republic of the Congo, 2013 Abbreviations: EC, emergency contraception; FP, family planning; IUD, intrauterine device; PEPFAR, US President's Emergency Plan for AIDS Relief.

Analysis of facility performance by type of support revealed that PEPFAR sites were more likely to be rated as 3 stars—suggesting readiness to provide family planning services—than other sites in Kinshasa (77.7% versus 56.9%, respectively). As shown in Figure 4, PEPFAR-supported sites were more likely than sites supported by traditional family planning partners (or those receiving no external support) to have every one of the 8 contraceptive methods in the method mix. However, PEPFAR-supported sites provided far fewer CYP on average (23.7) than did sites receiving support from traditional family planning organizations (61.3) but more than sites receiving no external support (15.8) (Table 5).

PEPFAR-supported sites were more likely to be ready to provide family planning services than other sites but delivered fewer CYP on average than sites receiving support from family planning organizations.

### Correlates of CYP in a Multivariate Model

As seen in the multivariate model (Table 6), 3 variables were associated with CYP: type of health facility, length of time in operation, and number of contraceptive methods available. Clinics generated higher CYP than hospitals and health centers by 65.3 and 61.5 units, respectively (*P*<.01). The longer facilities had been in operation, the higher the level of mean CYP (by 26.9 more CYP for those operating 4–6 years [*P*<.05] and 50.2 more CYP for those operating 7 or more years [*P*<.01]), as compared with the reference group of facilities in operation for 1 year or less. In terms of number of methods available at the facility and CYP, for each additional method available, CYP increased by almost 8 units (*P*<.01).

3 factors were associated with CYP: type of facility, length of time in operation, and number of contraceptive methods available.

**TABLE 6:** Multivariate Regression Analysis of Factors Associated With 3-Month CYP

	Mean CYP (SE)
Managing authority	
Government	1 [Reference]
NGO	19.65 (1.34)
Faith-based	12.34 (0.93)
Private	5.36 (0.40)
Other	9.55 (0.18)
Facility type	
Clinic	1 [Reference]
Hospital	-65.26 (2.79)**
Health center	-61.50 (2.86)**
Source of external support	
No support	1 [Reference]
FP implementing agency	13.23 (0.99)
PEPFAR	-18.91 (1.28)
No. of years in operation	
0–1	1 [Reference]
2–3	13.72 (1.10)
4–6	26.87 (1.97)*
≥7	50.19 (3.43)**
No. of days per week in operation	
1–3	1 [Reference]
4–6	19.16 (1.79)
7	3.49 (0.25)
Number of methods available	7.75 (3.06)**
Number of trained staff	3.45 (1.28)
Information system (yes/no)	8.06 (0.58)

Abbreviations: CYP, couple-years of protection; FP, family planning; PEPFAR, US President’s Emergency Plan for AIDS Relief; SE, standard error.

* *P*<.05, ** *P*<.01, *** *P*<.001

## DISCUSSION

Our experience in the DRC shows that smartphone technology can be applied effectively to conduct facility-based surveys, even in a low-resource setting with notable infrastructure constraints. In addition to significantly improving the timeliness of the data collected, the digital data collection methodology also improved data quality (with automated skip patterns and response constraints limiting the number of missed or invalid answers), facilitated monitoring and communication through the daily updates on the online server, reduced survey costs, and built valuable skills among data collectors and supervisors. Despite the initial investment into the smartphones, extra batteries, and power banks, expenses associated with ODK totaled less than the printing, shipping, and data entry costs typical of paper surveys. Moreover, the growing popularity of smartphone-based data collection among development agencies means that our partners in the DRC received credit for spearheading these initiatives in a severely resource-constrained environment and after several more rounds of digital data collection (including the PMA2020 program described below), the country’s public health programs now have a trained cohort of supervisors and data collectors who can rapidly and efficiently provide routine and strategic health information.

Facility-based surveys for family planning were conducted in 12 sub-Saharan African countries from 1989–97 by the Population Council under the Situation Analysis project.^13^ This model evolved into the Service Provision Assessment of the DHS, which has been carried out in some 10 African countries—most of which were conducted one time only in each country. With few exceptions (one being an article on Lesotho by Tuoane et al.^14^), few family planning facility-based surveys have been published in the peer-reviewed literature.^15^ However, with the advent of PMA2020, data on family planning SDPs will become available on an annual basis from at least 9 African countries.^5^

The major role played by the non-public sector in delivering family planning services highlights the longer-term challenge to the government of coordinating planning and budgeting. This analysis suggests a variation on the “total market approach” (whereby different sectors provide contraception for different segments of the population, based largely on ability to pay). However, it begs the question of what role the government can or should play in family planning programming going forward, especially given the current predominance of external organizations and funding. The government has become increasingly engaged in the issue of family planning.^2^ It needs to further pursue the objectives of the National Multisectoral Strategic Plan for Family Planning: 2014–2020 with emphasis on developing human resources, mobilizing financial resources, and procuring family planning products.

A key finding of this paper is the contribution of PEPFAR-supported family planning programming. The results of the 2013 survey demonstrated a readiness in PEPFAR-supported sites to provide services (as measured by the 3-star rating). However, the actual volume of services delivered (CYP) was lower than for sites supported by family planning implementing partners. This latter finding could reflect lack of interest, aptitude, and/or experience in family planning service delivery among staff trained primarily for HIV prevention and treatment. Alternatively, it may be a methodological artifact (the CYP data were collected for January–March 2013, during which point a number of PEPFAR facilities had just begun to integrate family planning with HIV). In fact, one-third of PEPFAR sites had been in operation for less than a year at the time of the 2013 survey (whereas only 15% of family planning sites were “new”). However, even if one compares only the sites in operation for at least 1 year, the mean CYP was higher among those supported by traditional family planning organizations (68) than among PEPFAR-supported sites (31) or no external support (21); data not shown in the tables. Of interest, the USAID Kinshasa Reproductive Health Advisor confirmed that the CYP data obtained in the 2013 survey for PEPFAR-supported sites were similar to the CYP numbers submitted by these same agencies to USAID (personal communication with Thibaut Mukaba, Family Planning/Reproductive Health Specialist, USAID/DRC, March 2015).

The case of Kinshasa can in no way be generalized outside Kinshasa. In particular, the findings could be very different in a country with high HIV prevalence, but they underscore the capacity of local facilities to scale-up family planning services if resources are made available, as was the case with PEPFAR funding in the DRC in 2013. However, in 2014 PEPFAR introduced a new strategy that places greater emphasis on treatment, and as a result family planning services will be discontinued at a number of sites previously supported with PEPFAR funding. These priority shifts will decrease access to family planning through health facilities in Kinshasa. Efforts are underway to identify new mechanisms for external support, with the withdrawal of PEFPAR funding for prevention activities.

When resources are made available, local facilities can scale-up services.

The facility-based surveys conducted in Kinshasa do not answer the question of what would be the ideal number of family planning facilities in Kinshasa. (A parallel paper not yet published does analyze the spatial distribution of sites in Kinshasa.) However, results on the number of sites and on the average number of methods available per site do indicate that there are almost 400 health facilities and more than 1,500 pharmacies in the city where potential clients could obtain contraceptive methods, if they had the desire and means to do so.

In short, the findings suggest that lack of physical access to contraceptive methods is not the defining reason for low modern contraceptive use in Kinshasa. In the 2015 PMA2020 survey, women aged 15–49 years not using contraception were more likely to give reasons other than service availability to explain their non-use: not at risk (44.7%), not married (39.8%), method or health-related concerns (19.3%), or opposition to use (11.9%); only 9 women (less than 1%) cited lack of knowledge/access to methods. Still, although not measured in these surveys, it is highly likely that other service-related factors also contribute to low service utilization, including quality of services, contraceptive stock-outs, and inconsistent pricing, among others.^16^

The survey findings suggest that lack of physical access to contraceptives is not the defining reason for low mCPR in Kinshasa.

### Value of the Survey Results

The findings from these facility-based surveys have contributed significantly to the increased momentum for family planning in the DRC since 2012 in multiple ways. Although we do not have concrete evidence demonstrating the link between the survey results and subsequent events, we believe the following activities can be plausibly linked to the survey work.

**Mobilization of implementing partners around a common objective of increasing contraceptive access and readiness.** Once the results from the 2012 survey were available, including static and interactive maps of SDPs throughout Kinshasa, the PNSR convened a meeting of the key donors for family planning in Kinshasa (USAID, UNFPA, and the Canadian Department of Foreign Affairs, Trade and Development) as well as implementing partners (listed in the Box) to review the findings. At this first meeting, the group, named the Kinshasa Family Planning Coalition, established the ambitious goal of increasing the percentage of 3-star sites in Kinshasa from 44% to 80%. The intervention to bring about this change was a series of quarterly meetings that focused on each of the 3 “stars” (range of methods, trained personnel, and functioning information system). The follow-up survey in late 2013 (then approximately 12 months away) would evaluate the extent of change. Although the follow-up survey showed an increase from 44.1% to only 63.3%, this measurable achievement in a relatively short period of time was highly motivational, and it created a new level of cohesion among family planning service providers in Kinshasa.^3^

**Development of an inventory of all family planning sites by implementing partner.** At the time of the 2012 survey, there was no central listing of family planning sites in Kinshasa, much less an inventory of which implementing partners supported which sites. Between the 2012 and 2013 survey, a spreadsheet was developed that showed every family planning facility listed by health zone and by implementing partner supporting that facility. If there had been a single managing authority responsible for all family planning sites (e.g., the Ministry of Health), this information would presumably exist. However, in a city where 10 different NGOs supported family planning service delivery without any formal coordination, this information linking family planning health facilities to specific implementing partners represented an essential step in defining the family planning supply environment. Moreover, it showed that some sites were supported by more than one partner organization (unbeknownst to them), while others had no supporting partner organization (which, once identified, could potentially be targeted to receive support in the future).

**Provision of feedback to implementing partners about their sites’ readiness to provide services and output.** Having linked family planning health facilities with implementing partners, the research team developed individual reports for each implementing organization. The report listed the name and address of the family planning facilities supported by each organization and provided information on the variables available from the 2013 survey: type of facility, managing authority, number of trained staff, and volume of each method in stock. Reports also gave the price that each facility charged for methods and the CYP measured for each facility. The director of the PNSR wrote a cover letter to each organization to reinforce the enhanced role of the PNSR in coordinating family planning activities in Kinshasa. Each implementing organization was free to use this information as it wished. Although we have no information on the extent to which organizations used these data for midcourse changes, these reports reflect the value of surveying the universe of family planning sites (instead of only a sample).

**Creation of heightened visibility for family planning in the DRC at the international level.** The static and interactive maps of family planning service delivery in Kinshasa were first presented publicly in November 2012 in Dar es Salaam, Tanzania, at a meeting titled “Using mobile technology to improve family planning and health programs.”^17^ Given that francophone African countries have traditionally lagged behind anglophone African countries in all aspects of family planning programming, the audience took note that the DRC was using this cutting-edge technology to improve the evidence base of its programming, and the DRC won the Innovation Award (personal communication with Thibaut Mukaba, Family Planning/Reproductive Health Specialist, USAID/DRC, November 2012). Heightened visibility can lead to increased donor investment, as has occurred for family planning in the DRC.

**Increase in donor investment in family planning.** Although one cannot establish a definitive causal effect, the growing interest among donors in supporting family planning in the DRC can be partially linked to the strong evidence base that is now available for family planning service delivery in Kinshasa. For example, in 2014 the David and Lucile Packard Foundation funded a major family planning initiative for community-based distribution in Kinshasa, which used the maps from the 2013 survey in identifying health zones (and even areas within health zones) underserved for family planning. The Bill and Melinda Gates Foundation, which had provided seed funding for establishing this evidence base, has subsequently funded additional family planning activity in Kinshasa and Kongo Central.

### Strengths and Limitations

Given the large increase in health facilities in Kinshasa reporting provision of family planning services between the 2012 and 2013 surveys (184 to 395), it is likely that the 2012 survey undercounted the number of sites. Specifically, in 2012 if the local health zone authorities informed the research team that a certain facility did not provide family planning, the research team did not visit that facility (which probably contributed to the undercount). However, as detailed in the results section, at least part of the increase can be attributed to the addition of new sites by PEPFAR-supported partners and by established family planning implementing agencies.

The 2012 and 2013 surveys were designed to obtain a few key variables from the universe of health facilities offering family planning in the 35 health zones of Kinshasa (in contrast to conducting a more comprehensive assessment of each facility based on a sample of family planning sites, as does the PMA2020 SDP module, which was conducted in Kinshasa in 2014 and 2015 and will continue forward in the coming years). In particular, because the 2012 and 2013 surveys collected relatively little data on each health site, the “3-star” rating system is based on only 3 variables (availability of methods, trained personnel, and information system).

By conducting surveys of “the universe” versus a sample of family planning sites in 2 consecutive years, we better appreciate the strengths and limitations of the 2 approaches. For example, the 2013 survey (“universe” approach) was extremely valuable for programmatic purposes. It provided site-specific information that could be fed back to implementing organizations for midcourse corrections and that could be used to answer such questions as which sites carried the implant in 2013 and how those sites were distributed across Kinshasa. Moreover, it captured a full picture of provision of family planning services in all health zones of Kinshasa. By contrast, the PMA2020 SDP survey yielded a sample of SDPs (including pharmacies, not included in the 2013 survey), which permits tracking of progress over time; moreover, PMA2020 allows for linking population-based data on contraceptive use among women residing in each enumeration area to data on access and service readiness at the facilities in the same enumeration areas.

## CONCLUSION

This article forms part of an iterative series of quantitative and qualitative studies to understand the low mCPR in Kinshasa, DRC, and to inform family planning programming. It serves as an important reminder that physical access alone to health care services is just one part of the supply/demand equation. In the severely resource-constrained environment that is Kinshasa, the very existence and physical access to family planning SDPs is a necessary but not sufficient condition to the effective use of these services. Future research on contraceptive demand will yield further insights into the complex set of supply and demand factors that determine mCPR in Kinshasa, including financial, cultural, and social barriers.

## Notes

**Peer Reviewed**
